# Drought Tolerance Conferred to Sugarcane by Association with *Gluconacetobacter diazotrophicus*: A Transcriptomic View of Hormone Pathways

**DOI:** 10.1371/journal.pone.0114744

**Published:** 2014-12-09

**Authors:** Lívia Vargas, Ailton B. Santa Brígida, José P. Mota Filho, Thais G. de Carvalho, Cristian A. Rojas, Dries Vaneechoutte, Michiel Van Bel, Laurent Farrinelli, Paulo C. G. Ferreira, Klaas Vandepoele, Adriana S. Hemerly

**Affiliations:** 1 Laboratório de Biologia Molecular de Plantas, Instituto de Bioquímica Médica Leopoldo de Meis, Universidade Federal do Rio de Janeiro, Cidade Universitária, Rio de Janeiro, RJ, Brazil; 2 Universidade Federal da Integração Latino-Americana, Foz do Iguaçu, PR, Brazil; 3 Department of Plant Systems Biology, VIB, Gent, Belgium; 4 Department of Plant Biotechnology and Bioinformatics, Ghent University, Gent, Belgium; 5 Fasteris SA, Plan-les-Ouates, Switzerland; Purdue University, United States of America

## Abstract

Sugarcane interacts with particular types of beneficial nitrogen-fixing bacteria that provide fixed-nitrogen and plant growth hormones to host plants, promoting an increase in plant biomass. Other benefits, as enhanced tolerance to abiotic stresses have been reported to some diazotrophs. Here we aim to study the effects of the association between the diazotroph *Gluconacetobacter diazotrophicus* PAL5 and sugarcane cv. SP70-1143 during water depletion by characterizing differential transcriptome profiles of sugarcane. RNA-seq libraries were generated from roots and shoots of sugarcane plants free of endophytes that were inoculated with *G. diazotrophicus* and subjected to water depletion for 3 days. A sugarcane reference transcriptome was constructed and used for the identification of differentially expressed transcripts. The differential profile of non-inoculated SP70-1143 suggests that it responds to water deficit stress by the activation of drought-responsive markers and hormone pathways, as ABA and Ethylene. qRT-PCR revealed that root samples had higher levels of *G. diazotrophicus* 3 days after water deficit, compared to roots of inoculated plants watered normally. With prolonged drought only inoculated plants survived, indicating that SP70-1143 plants colonized with *G. diazotrophicus* become more tolerant to drought stress than non-inoculated plants. Strengthening this hypothesis, several gene expression responses to drought were inactivated or regulated in an opposite manner, especially in roots, when plants were colonized by the bacteria. The data suggests that colonized roots would not be suffering from stress in the same way as non-inoculated plants. On the other hand, shoots specifically activate ABA-dependent signaling genes, which could act as key elements in the drought resistance conferred by *G. diazotrophicus* to SP70-1143. This work reports for the first time the involvement of *G. diazotrophicus* in the promotion of drought-tolerance to sugarcane cv. SP70-1143, and it describes the initial molecular events that may trigger the increased drought tolerance in the host plant.

## Introduction

Sugarcane (Saccharum spp.) has become an important bioenergy crop worldwide. Its capability to store sucrose as a primary energy source, instead of more complex compounds as starch, proteins or lipids, makes its use for energy production easier. Statistical data from the Food and Agriculture Organization of the United Nations indicate that sugarcane production covered, in 2012, more than 26 million hectares worldwide, with a global production of 1,8 billion tons, representing a 30% of growth since the beginning of the century. Nowadays, Brazil stands as world's first country in sugarcane harvesting and production, being alone responsible for 40% of the global production (http://faostat3.fao.org/). Given the growth of sugarcane industry worldwide, demands for higher sugarcane yield per hectare have been increasing to lower production costs, especially in areas with adverse conditions such as drought and cold. As water availability is the major limiting factor for sugarcane productivity [1], studies that lead to an increase of sugarcane drought tolerance are needed to provide tools to allow sugarcane plantation in drier regions. This is one of the greater challenges for the sustainable expansion of sugarcane production that is being carried out in Brazil since 2007 [2].

As most grasses, sugarcane established during its evolution an efficient interaction with particular types of microorganisms. Plant-growth-promoting-bacteria (PGPB) can offer several benefits to host plants such as increase in biomass, promotion of plant development [3], [4], pathogen defense [5]–[7] and tolerance to abiotic stresses, including drought [8]–[10]. Some PGPB can also transfer fixed nitrogen to plants, in a process called Biological Nitrogen Fixation (BNF) [11]. The diazotrophic PGPB comprise many species, including *Gluconacetobacter diazotrophicus* and facultative endophytes as *Azospirillum*, among others [12]. The use of inoculants containing nitrogen-fixing bacteria can generate large benefits to farmers, for example by decreasing nitrogen fertilizer expenses and reducing losses by drought or diseases. However, these gains depend on cultivars able to take full advantage of the benefits generated by bacterial associations. Previous studies reviewed plant drought tolerance promoted by several types of microorganism interactions such as fungi [13], endophytic and rhizospheric bacteria [14] and rhizobium interaction [15]. Some works specifically described drought tolerance improved by *Azospirillum*, a facultative endophyte diazotrophic bacteria, in monocots [16]–[18] and in dicots [19]. However, little is known about the molecular pathways involved in the drought tolerance promoted by beneficial endophytic diazotrophic bacteria that colonize sugarcane. Besides drought tolerance, the use of diazotrophic bacteria in crop production can also help minimizing the use of nitrogen fertilization [20]. As potential groundwater pollutant, nitrogen fertilization is a major environmental concern related to agriculture practice [21]. Thus, a better understanding of the interaction between host plant and diazotrophic bacteria may favor the emergence of new technologies that enable more sustainable use of natural resources in agricultural production. With the purpose to efficiently use water in sugarcane crop production, enlightening the molecular mechanism involved in drought tolerance promoted by plant-diazotroph interaction might be a step forward to bring agriculture to a more sustainable level.

Plant responses to drought involve a complex network of signaling mechanisms, regulating several different physiological and biochemical processes [22]–[24], and responses could be different depending on plant species or even cultivars [25]. Hormone regulation is known to have an important role in plant responses to abiotic stresses. Because it regulates diverse processes in plants, hormone signaling can induce some levels of stress tolerance [26]. Abscisic acid (ABA) and ethylene (ET) are stress responsive hormones that play important roles in drought sensing and response [27], [28]. The drought responsive gene expression can be regulated by ABA-dependent or ABA-independent pathways, mainly through Transcription Factors (TFs) family ABRE-binding protein/ABRE-bing factors (AREB/ABF) or DRE/CRT-binding protein (DREB), respectively [29]. The AREB/ABF TFs are members of the Basic Leucine Zipper (bZIP) superfamily of TFs with a binding motif to ABA-responsive element (ABRE), and the DREBs are members of APETALA2 (AP2)/ethylene-responsive element binding factor (ERF) with a binding motif to dehydration-responsive element/C-repeat (DRE/CRT), which are known to be involved in abiotic stress responses in Arabidopsis and Grasses [30].

In this work, responses of sugarcane-diazotrophic bacteria association were characterized during drought stress. For this purpose, a water depletion assay of the interaction between the sugarcane cultivar SP70-1143 and the diazotroph *G. diazotrophicus* strain PAL5 was performed. Sugarcane cv. SP70-1143 responds efficiently to diazotrophic bacteria association, whose contribution of BNF is estimated to be able to reach levels up to 70% [11], [31]. SP70-1143 was widely cultivated in the mid-80s in Brazil and today participates as progenitor of new improved varieties [32], which highlights the importance this cultivar still has in Brazilian agriculture. On the other hand, *G.diazotrophicus* is known to establish beneficial interactions with sugarcane, providing a good model system for associations between monocots and diazotrophic bacteria [33]. The results indicate that under our experimental conditions, sugarcane cv. SP70-1143 plants colonized with *G. diazotrophicus* became more tolerant to drought stress than non-inoculated plants. Higher levels of *G. diazotrophicus* colonization were quantified in inoculated plants after water deficit, compared with plants growing in normal watering conditions. To better understand the early molecular responses promoted by diazotroph-sugarcane interactions in plants submitted to drought stress, especially the hormone signaling responses, differential transcriptomes of shoots and roots of cv. SP70-1143 inoculated by *G. diazotrophicus* submitted to water deficit were generated by RNA-Seq. The results showed that SP70-1143 plants free of diazotrophs respond in a classical way to water deficit, activating well-known drought-responsive markers. Nevertheless, bacteria's colonization suppresses in the host plant several of the drought stress responses.

## Results and Discussion

### Colonization of sugarcane cv. SP70-1143 by *G. diazotrophicus* confers tolerance to water deficit

**Figure 1:**
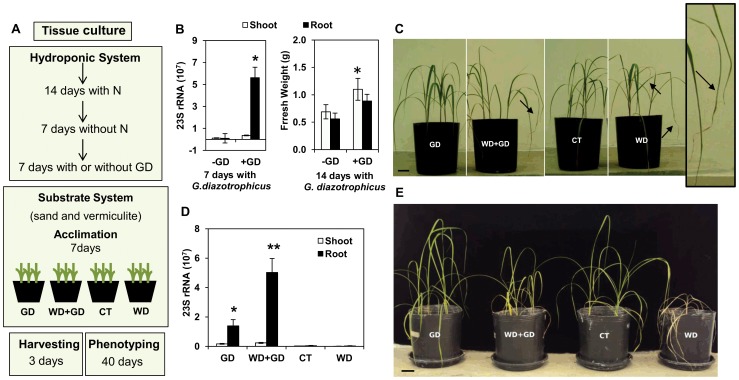
Water deficit assay of sugarcane cv. SP70-1143 colonized with the beneficial endophytic diazotrophic bacteria *G. diazotrophicus* strain *PAL5*. (a) Simplified pipeline for water deficit assay. (b) left panel shows qRT-PCR quantification of sugarcane colonization by *G.diazotrophicus* 7 days after inoculation in hydroponic solution. Bacterial 23S rRNA levels are presented relative to rice 28 S rRNA levels. Right panel shows root and shoot fresh weight measurements 14 days after inoculation and 1 day before treatments. (c) Phenotype of sugarcane cv. SP70-1143 inoculated or not with *G. diazotropicus*, 3 days after withholding water. Senescent edges are indicated by arrows and are shown in the amplified image from a WD+GD plant. (d) qRT-PCR quantification of sugarcane colonization by *G.diazotrophicus* after 3 days under water deficit assay. Bacterial 23S rRNA levels are presented relative to rice 28 S rRNA levels. (e) Phenotype of sugarcane cv. SP70-1143 inoculated or not with *G. diazotropicus*, after 40 days withholding water. Bar = 5 cm. Error bars indicate standard error of the mean. Asterisk mark statistical significance between GD-R vs CT-R (*p<0.05) and WD+GD-R vs GD-R (**p<0.01), performed by statistical t-test (unpaired).

In order to investigate the early molecular and biochemical responses to water deficit of sugarcane cv. SP70-1143 colonized by the beneficial diazotrophic bacteria *G. diazotrophicus*, four different plant treatments were analyzed, following the pipeline presented in Figure 1A: i) plants in association with *G. diazotrophicus* and under normal watering conditions (GD); ii) in association with *G. diazotrophicus* and under water deficit (WD+GD); iii) non-inoculated and under normal watering conditions (CT); iv) non-inoculated and under water deficit (WD).

To confirm that plants were associated with *G. diazotrophicus* in a beneficial way before performing water deficit treatments, controls of bacteria colonization and of the increase in plant biomass were carried out. As shown in Figure 1B, quantification of *G.diazotrophicus* colonization was measured by qRT-PCR of ribosomal RNA (23S rRNA). Ribosomal RNA is considered a constitutive gene, and it is widely used to detect the presence of target microorganisms in a variety of samples [34]. The results showed that plants were highly colonized by bacteria after 7 days of inoculation in hydroponic solution. Interestingly, bacteria colonized roots in higher numbers than shoots when the hydroponic system was used for plant inoculation and growth. To be certain that bacteria were promoting benefits to host plant, the fresh weight of roots and shoots was evaluated 14 days after inoculation, confirming the plant growth promotion effect of *G. diazotrophicus* one day before treatments (Figure 1B).

A second type of analysis was conducted to define the period of water deficit treatment suitable for the investigation of early molecular and biochemical responses of cv. SP70-1143 to water deficit. As physiological modifications are the first responses of plants to overcome water deficit, phenotypic analysis of SP70-1143 plants were monitored along water deficit condition. As shown in Figure 1C, plants in treatments WD and WD+GD started showing visible signals of stress, such as leaves with senescent edges, 3 days after withholding watering, and leaf rolling was not observed. No significant difference was found in fresh weight after 3 days of treatments (not shown). Sugarcane cultivars with contrasting tolerance to drought, as RB 867515 (higher tolerance) and RB855536 (low tolerance), showed both leaf rolling and senescence after the second day of stress [35], suggesting that SP70-1143 cultivar responds in a similar timing, although possibly using different strategies to avoid drought.

To answer whether water conditions would affect plant colonization by *G. diazotrophicus*, qRT-PCR of ribosomal RNA (23S rRNA) was performed in order to detect *Gluconacetobacter* genera in all treatments after 3 days under water deficit. As shown in Figure 1D, relative expression of *Gluconacetobacter* 23S rRNA indicated the presence of high numbers of bacteria in inoculated root samples compared to non-inoculated samples, validating the successful plant colonization. Interestingly, inoculated root samples of SP70-1143, after 3 days under water deficit (WD+GD), showed 23S rRNA levels 3-fold higher than inoculated roots with normal watering conditions (GD) (Figure 1D). By contrast, inoculated shoot samples subjected or not to water deficit (WD+GD or GD) did not show a significant expression of 23S rRNA, indicating that under these experimental conditions, the bacteria do not colonize shoot tissues efficiently. The data suggests that drought stress could lead to higher colonization rates by *G. diazotrophicus* in root tissues of sugarcane cultivar SP70-1134. As the aim of this work is to address the early responses to drought in plants colonized with *G. diazotrophicus*, samples 3 days after treatments were chosen to be further analyzed.

Next, to investigate the impact of a prolonged water deficit to sugarcane cv. SP70-1143 when associated with *G. diazotrophicus*, treatments were maintained for 40 days (Figure 1E). While non-inoculated SP70-1143 plants died after a month withholding watering, plants inoculated with the beneficial diazotroph survived, albeit with visibly shorter shoots compared to inoculated plants normally watered (Figure 1E). As the analysis showed high levels of *G. diazotrophicus* colonization in plants under water deficit (Figure 1D), the data indicates that sugarcane cv SP70-1143 plants colonized with *G. diazotrophicus* become more tolerant to drought stress than non-inoculated plants.

Drought tolerance promoted by the interaction of diazotrophs, such as *Azospirillum brasilense*, and grasses, such as wheat, maize and rice, were already reported. In wheat, *Azospirillum* inoculation mitigated at least 16% of the negative effects provoked by drought on grain yield [16]. When *A. brasilense* are genetically modified to over-accumulate the osmoprotectant trehalose, inoculation with these bacteria can enhance the percentage of maize survival to drought compared to the results obtained with a wild type strain [17]. In rice plants it was also shown that a microbial consortium between an arbuscular mycorrhizal fungus and *Azospirillum* can improve growth and tolerance to drought more than each one separately [18]. In common, these studies indicate that *Azospirillum* and other plant growth-promoting microorganisms can induce stress tolerance by improving plant water status trough the enhancement of protective compounds such as trehalose, proline and ascorbate.

The *G. diazotrophicus* strain PAL5 investigated in this work is considered an obligatory endophyte and has particular molecular and biochemical features, as compared to other diazotrophic bacteria [36]. *Azospirillum* genus is constantly addressed as facultative endophyte, and possibly interacts differently with host plants than *G. diazotrophicus*. In our experimental conditions, the obligatory endophyte *G. diazotrophicus* colonized sugarcane cv. SP70-1143 plants under water deficit, and these plants became more tolerant to drought stress than non-inoculated ones (Figure 1E).

### High-throughput mRNA-sequence analysis

#### RNA-sequencing and *de novo* assembly

Although several published results show that microorganisms improve abiotic stress tolerance in dicots and monocots [13]–[15], little is known about how they affect plant molecular and biochemical responses. In an attempt to improve our understanding of the role of diazotrophic bacteria to drought tolerance in sugarcane, mRNA libraries of root and shoot of plants from four experimental conditions, after 3 days with or without withholding watering, were generated and sequenced using RNA-seq. The adopted abbreviations are shown in Table 1.

**Table 1:** Abbreviations used for 8 sample types and dataset comparisons.

**experimental condition**	CT	normal watering
	GD	bacteria association and normal watering
	WD	water deficit
	WD+GD	bacteria association and water deficit
**tissue type**	R	Root
	S	Shoot
**dataset comparisons (** ***short name*** **)**	GD *vs* CT	plant response to association with bacteria (*Bacteria*)
	WD *vs* CT	plant response after 3 days under water deficit (*Drought*)
	WD+GD *vs* WD	plant response to water deficit when associated with bacteria (*Bacteria&Drought*)*

* dataset after subtract loci that are equaly regulated by GD *vs* CT and WD *vs* CT.

**Figure 2:**
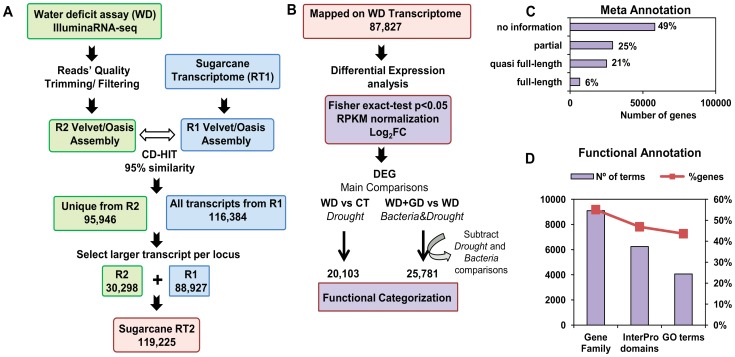
Simplified pipeline of the construction and analyses of the sugarcane reference RT2. (a) Construction of the sugarcane reference transcriptome RT2. The total numbers of transcripts or loci/gene quantified in each step is shown. (b) Generation of differentially expressed transcriptomes. Numbers represent a total of up and downregulated DEG in each dataset, details are presented in Table 4. (c) and (d) represent TRAPID-based analysis of (c) sequence length meta annotation and (d) number of Gene families, Protein domains and GO terms found in RT2, and the percentage of genes that were annotated in at least one of the tree functional categories.

A summary of the RNA-seq construction and computational analysis is presented in Figure 2A,B. Between 19 and 26 million 100 bp single-end reads were obtained from each of the eight cDNA libraries derived from the sugarcane SP70-1143 samples. The sequences of low quality were removed using quality trimming and quality filtering. Table 2 shows the number of reads retained after each processing step, which were used to perform *de novo* assembly. In order to generate a sugarcane reference transcriptome suitable to this study, two assemblies were performed, both using the VELVET transcriptome assembler [37] in combination with OASES [38]. The first assembly, called R1, was the sequencing result from a previous sugarcane transcriptome experiment, using root and shoot tissues of two sugarcane genotypes, with different efficiencies of BNF: SP70-1143 (high BNF) and Chunee (low BNF). The second assembly, R2, was performed during this work, using the sequencing result of the eight cDNA libraries derived from the four different treatments. A reference transcriptome was obtained combining R1 and R2. To avoid redundancy, CD-HIT [39] was used to search for similar sequences between both assemblies, with a threshold of 0.95. A total of 95,946 unique transcripts were detected from R2, and combined to 116,384 transcripts from R1 (Figure 2A). Between 50 to 55% of mapped reads matched several positions on the RT2, indicating a high percentage of non-unique read mappings in each library. In this case, the read mapping tool was assigning each read randomly to a transcript. Due to this random effect, it was possible to infer mistaken differences in expression for transcripts of the same locus. To avoid this issue, the longest transcript was selected to represent one locus. Using one transcript per locus, these reads were all used to quantify a single sequence per locus. With this procedure, 30,298 loci were obtained from R2 and 88,927 from R1, generating a total of 119,225 sequences in the Sugarcane Reference Transcriptome (RT2). Transcriptome sequences were evaluated against Viridiplantae proteins in the PLAZA 2.5 database using a BLASTX search [40]. An E-value cutoff threshold of 1e-05 was considered to define a significant hit. A total of 60,376 sugarcane sequences produced a hit against any plant. Of these, 54,747 produced a hit against a monocot proteome database (*Oryza sativa* ssp indica, *Oryza sativa* ssp. japonica, *Sorghum bicolor*, *Brachypodium dystachion* or *Zea mays*). A group of 58,849 loci did not map with plant databases. Since their sequences are mostly very short, these loci are probably parts of real transcripts that could not be merged in the assembly. Since it has been demonstrated that bacterial RNA can be found as polyadenlated in transcriptome sequencing studies [41], a BLASTN search for *G. diazotrophicus* sequences was carried out in the non-plant loci dataset (E-value cutoff 1e-05) and no hits were found.

**Table 2:** Summary of Illumina transcriptome sequence data and quality control.

Sample	Raw reads	Trimmed reads	Filtered reads	% *
CT-S	19,124,841	19,115,843	17,924,240	93.72
WD-S	20,991,631	20,980,784	19,758,556	94.13
GD-S	25,947,454	25,935,398	24,318,355	93.72
WD+GD-S	23,292,388	23,275,855	21,872,317	93.90
CT-R	19,851,601	19,839,582	18,484,006	93.11
WD-R	20,578,612	20,566,524	19,187,334	93.24
GD-R	20,867,493	20,856,633	19,430,244	93.11
WD+GD-R	21,582,269	21,569,888	20,067,616	92.98

* percentage of reads left after trimming and filtering.

#### Differential analysis and functional annotation of sugarcane transcriptome

To analyze mRNA expression in each locus, BWA mapping software [42] was used to map the reads against the complete RT2. From the total number of reads generated by RNA-seq, after the quality filtering and trimming, the ones mapped to the reference transcriptome represented a total of 87,827 loci and an average of 72% in shoot samples and 39% in root samples. A general view of the raw data of all eight RNA-seq is presented in Table 3. Despite the low percentage of mapped reads observed in roots libraries, the number of loci with at least one read was very close between all roots and shoots libraries. However, compared to root libraries, shoot libraries still had more loci with reads higher than 1000. To select differential expressed loci, read counts were normalized as RPKM.

**Table 3:** Summary of reads and genes (loci) mapped in each generated library.*

Description	CT-S	WD-S	GD-S	WD+GD-S	CT-R	WD-R	GD-R	WD+GD-R
**Total reads** a	17,924,240	19,758,556	24,318,355	21,872,317	18,484,006	19,187,334	19,430,244	20,067,616
**Mapped reads** b	12,425,731	14,659,253	17,129,829	16,136,699	6,558,585	10,398,111	8,362,238	5,654,445
**Mapped bases (Mb)**	1,209	1,432	1,670	1,575	613	999	792	518
**Loci with at least one read** c	74,305	74,143	76,278	73,874	65,803	73,587	69,857	59,582
**Loci with>1000 reads** c	1,886	2,466	2,862	2,899	501	1,358	836	308

* Bases in deletions or insertions were not counted here.

a Number of reads obtained after Illumina sequencing and filtering.

b Number of reads mapped against reference transcriptome.

c Number of loci from a total of 87,827 loci mapped.

Pairwise comparisons were made between libraries of each tissue, to calculate changes in expression (Log_2_FC) of individual loci (hereafter considered as genes). Upregulated and downregulated differentially expressed genes (DEG) were selected by Fisher exact-test (p<0.05) and by having at least 2-fold difference between the library of interest and the selected control (see Material and Methods). The comparisons GD vs CT, WD vs CT, WD+GD vs WD identified genes related, respectively, to i) the association between plant-bacteria in normal water conditions, ii) the plant responses after 3 days under water deficit and iii) the plant response to water deficit when associated with bacteria. To better select genes related to plant response to water deficit only when associated with bacteria, all loci equally regulated by GD *vs* CT and WD+GD *vs* WD, and by WD *vs* CT and WD+GD *vs* WD were subtracted from WD+GD *vs* WD comparison (Figure S1), leaving a dataset of genes uniquely regulated by bacteria during water deficit. As summarized on Table 1, the DE datasets were called, i) *Bacteria*, for GD *vs* CT comparison, ii) *Drought*, for WD *vs* CT comparison, and iii) *Bacteria&Drought*, for WD+GD *vs* WD comparison. In this work, the *Bacteria* dataset was analyzed as a control for the *Bacteria&Drought* dataset. Figure 2B shows the overall steps to select DEG after the mapping. A summary of the identified genes differentially regulated in the three datasets is presented in Table 4. The subtracted *Bacteria&Drought* comparison showed an elevated number of DEG in roots compared to the *Drought* dataset. For both roots and shoots, more than 12 thousand genes seemed to be regulated uniquely in plant response to water deficit when associated with *G. diazotrophicus*. It is interesting to note that both the *Drought* and the *Bacteria&Drought* datasets showed a great number of common genes oppositely regulated (Table 4). This result reveals that SP70-1143 responses to water deficit could be, in some ways, inversely regulated when SP70-1143 interacts with *G. diazotrophicus*.

**Table 4:** Overall summary of the differentially regulated genes in Sugarcane SP70-1143 in the tree datasets comparisons.

Dataset comparison	N° of DE genes		Up-regulated		Down-regulated	
	R	S	R	S	R	S
*Bacteria*	2,628	2,671	1,451	1,100	1,177	1,571
*Drought*	5,465	14,638	3,334	6,455	2,131	8,183
*Bacteria&Drought*	12,730	13,051	2,109	6,059	10,621	6,992
*Common to *Bacteria* and *Bacteria&Drought*	180	282	42	56	50	119
**Common to *Drought* and *Bacteria&Drought*	1,241	3,053	38	369	2	429
Only *Bacteria*	2,448	2,389	1,354	1,010	1,094	1,379
Only *Drought*	4,224	11,585	2,548	4,782	1,676	6,803

*55 (root) and 34 (shoot) transcripts are down in *Bacteria&Drought* and up in *Bacteria*; 33 (root) and 73 (shoot) transcripts up in *Bacteria&Drought* and down in *Bacteria*.

**748 (root) and 1,304 (shoot) transcripts down in *Bacteria&Drought* and up in *Drought*; 453 (roots) and 951 (shoots) transcripts up in *Bacteria&Drought* and down in *Drought*.

A reference genome, gene models and functional annotations are still not available for sugarcane. Therefore, to infer gene functions for the sugarcane transcriptome, it was used the TRAPID tool [43] and functional annotation based on best BLAST hit searches against *Arabidopsis thaliana* and *Oryza sativa* protein databases. TRAPID is a user-friendly web tool that allows functional and comparative analyses for *de novo* transcriptome data sets. The RT2 was loaded into TRAPID and processed for sequence similarity searches against reference monocot proteins and gene families (GF). From the total of 119,225 sequences, with an average sequence length of 550.9 bp, 27% presented a full-length or quasi full-length transcript, according to TRAPID meta-annotation analysis (Figure 2C). TRAPID found a total of 9,102 GF, 6,244 InterPro domains and 4,065 GO terms in the RT2, which included 55.1% genes in GF, 46.9% with protein domain and 43.6% with GO terms (Figure 2D). This data shows that it was possible to infer functions for at least 50% of the genes from the total RT2, despite the presence of many partial gene sequences.

**Figure 3:**
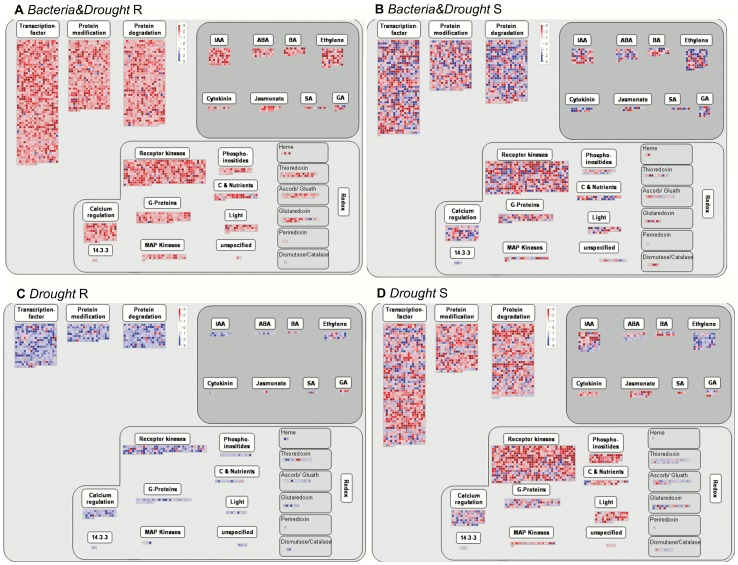
MapMan overview of differentially expressed genes in sugarcane SP70-1143 colonized or not with *G. diazotrophicus* and subjected to water deficit. MapMan ‘regulation overview’ of differentially expressed genes (DEG) from roots and shoots in two dataset comparisons: *Bacteria&Drought* and *Drought*. Blue stands for upregulated and red stands for downregulated loci. Only differentially expressed genes are shown, with p<0.05 and at least 2-fold difference from control.

Trough best BLAST hit searches against *A. thaliana* and *O. sativa* protein sequences, a functional MapMan mapping for putative sugarcane genes was created. The functional mapping generated a reference file that was uploaded on MapMan software [44] in order to visualize diagrams of metabolic pathways and other processes in the datasets. The functional contribution of each assembly, R1 and R2, in the final generation of RT2 is presented in Figure S2. The major contribution of R2 that is common to all tree datasets, in both roots and shoots, was seen in protein (3–6%), RNA (2–5%), signaling (2–3%), development (1–2%), transport (1–2%) and stress (1–2%) classes. The overall expression profiles of genetic regulation between roots and shoots in a same comparison, observed trough MapMan viewer, were strongly different (Figure 3), despite the number of DEG being very similar between root and shoot comparisons, except in *Drought* (Table 4). This result supports the empirical knowledge that plant tissues respond differently to environmental cues. Also, the fact that only in *Drought* the numbers of DEG are much higher in shoots than in roots might suggest that, in SP70-1143 plants under drought condition, shoots are more exposed to transcription regulation than roots, which could be related to evidences that shoot growth is more responsive to water deficit than root [45], [46].

### Transcriptional profiling of sugarcane cv SP70-1143 free of diazotrophic endophytes and under water deficit

Sugarcane is naturally colonized by diverse types of diazotrophic bacteria [47]. As very little is known about the molecular responses to water deficit of sugarcane plants free of diazotrophic bacteria, a drought transcript profile was first determined in diazotroph-free sugarcane cv.SP70-1143 plants. Next, DE transcripts organized in different functional categories were identified and analyzed in the *Drought* dataset.

#### Drought molecular markers in sugarcane under water deficit

**Figure 4:**
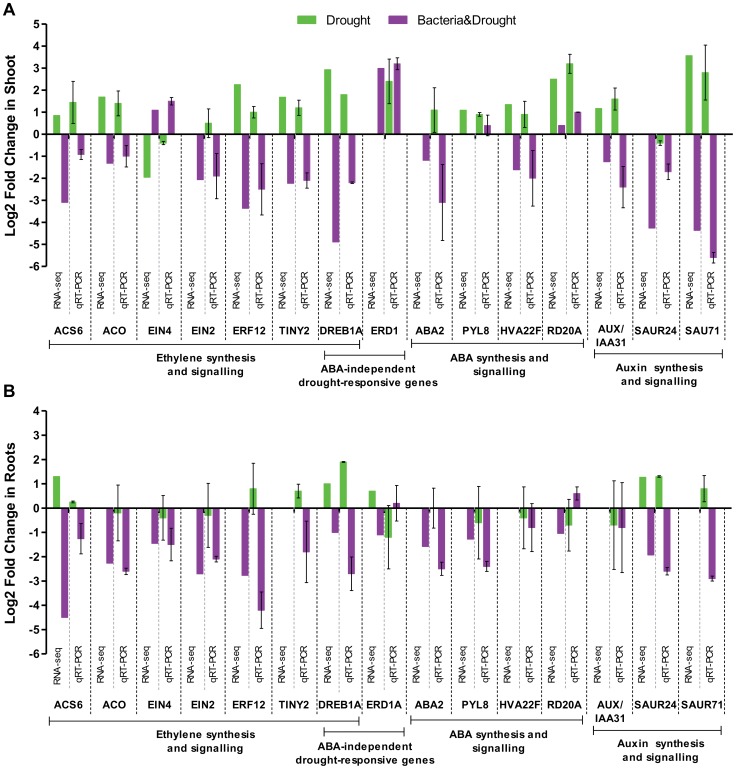
Validation of differentially expressed genes by qRT-PCR analysis compared to RNA-seq analysis. Pattern of expression of stress-responsive genes in (a) shoots and (b) roots of *Drought* and *Bacteria&Drought* datasets. qRT-PCR results are presented as the ratio of expression of each gene (relative to *28S rRNA*) in the treatments WD or WD+GD, compared to controls CT or WD respectively, and transformed in log2. Each biological replicate represents a pool of tree plants. Error bars indicate the standard error of the mean (n = 2).

Distinct plant species and genotypes can exhibit different physiological and developmental responses to water deficit [25]. Thus, to confirm on a molecular level that SP70-1143 plants were under drought stress 3 days after withholding watering, well established drought molecular markers were searched for in the *Drought* DEG dataset. Some of the most referenced gene markers that are generally regulated in abiotic stresses, i.e. the TFs ERF (Ethylene Response Factor, subfamily of ERF/AP2), CBF/DREB (Dehydration Response Element, subfamily of ERF/AP2), ERD (Early Responsive to Dehydration), RD (Response to Dehydration) and genes related to ABA pathway [27], [28], [48], were annotated in SP70-1143 under water deficit (Table 5). The expression pattern of the drought molecular markers ERD15 [49], DREB1A/CBF3 and DREB1B/CBF1 [50], [51] were up-regulated in both roots and shoots under water depletion (Table 5). qRT-PCR analysis also showed increased mRNA levels of ERD1 in shoots subjected to drought (Figure 4A). ERD1 is known to be induced not only in response to dehydration by an ABA-independent manner but also in the activation of senescence [23]. Members of DREB subfamily, on the other hand, can regulate drought responses by both ABA-dependent and independent pathways [50], [51]. DREB1A/CBF3 is known as a key regulator for cold and drought responses [52] and qRT-PCR analysis showed that its mRNA levels were induced in roots and shoots of SP70-1143 plants 3 days after withholding watering, validating the RNA-seq data (Figure 4). Some drought molecular markers involved in ABA signaling and response were present only in shoot tissues, as was the case of putative transcripts of HAI3/PP2C and HVA22E [53], [54]. HVA22 is a unique ABA/stress-induced protein, and it is known to be highly induced in leaves by ABA and drought [55]. The dehydration-responsive genes RD22 and RD20A were also upregulated in the *Drought* DEG dataset. They are known to respond to stress in an ABA-dependent manner - RD22 trough MYC and MYB recognition sites as *cis*-Acting regulatory elements and RD20A trough ABRE [23], [56], [57]. Corroborating the expression pattern described for RNA-seq analysis, qRT-PCR demonstrated an increase of RD20A and of HVA22F (an homologue to HVA22E) mRNA levels in shoots subjected to water deficit (Figure 4A). Altogether, the expression profile of drought molecular markers confirmed that SP70-1143 plants were under drought stress 3 days after withholding watering.

**Table 5:** Genes known to be upregulated in response to water deprivation and their expression in Drought dataset comparison for root and shoot tissues.

			*Drought*	
ID Reference	Mapman Category	Description	Root	Shoot
r1_l20127_t1	ABA synthesis	ABA1/ZEP	1.0	ns
r1_l8751_t1	ABA synthesis	NCED3	1.5	ns
r2_l7450_t18	ABA synthesis	NCED3	1.1	ns
r1_l58358_t1	ABA response	HVA22E	ns	1.0
r2_l11029_t7	ABA response	HVA22E	ns	1.2
r1_l34217_t1	postranslational modification	HAI3/PP2C	ns	1.2
r2_l20385_t22	postranslational modification	HAI3/PP2C	ns	1.1
r1_l12936_t1	regulation of transcription	ERF3	1.8	1.6
r1_l2576_t1	regulation of transcription	ERF3	ns	1.2
r1_l3102_t3	regulation of transcription	ERF7	1.0	1.1
r1_l2938_t3	regulation of transcription	ERF7	1.5	1.8
r2_l11720_t11	regulation of transcription	ERF7	1.4	1.6
r1_l1408_t2	regulation of transcription	CBF3/DREB1A	2.1	3.0
r1_l3179_t1	regulation of transcription	CBF3/DREB1A	3.0	2.3
r1_l6917_t2	regulation of transcription	CBF3/DREB1A	1.0	2.9
r1_l88_t1	regulation of transcription	CBF3/DREB1A	2.3	1.6
r2_l4653_t10	regulation of transcription	CBF3/DREB1A	3.6	3.2
r2_l16796_t3	regulation of transcription	CBF1/DREB1B	3.8	3.5
r1_l15430_t1	abiotic stress	ERD15	2.4	ns
r1_l3925_t2	abiotic stress	ERD15	ns	1.0
r1_l4012_t1	abiotic stress	RD20A	ns	2.5
r2_l8437_t21	abiotic stress	RD20A	ns	2.5
r1_l56448_t1	abiotic stress	RD22	ns	1.1
r2_l1357_t15	abiotic stress	RD22	ns	1.2
r1_l1866_t11	development/unspecified	SNAC1/NAC67	ns	1.7
r1_l18628_t1	not assigned/unknown	Dehydrin	2.3	2.9

Numbers show the log_2_ fold change of DE genes.

#### General expression pattern of sugarcane under water deficit

Next, functional enrichment analysis tools were applied to trace a more general water deficit profile in non-inoculated SP70-1143 plants. The characterization of enriched classes of RNA-seq data was performed with two complementary methods: PageMan gene set enrichment analysis and Gene Ontology (GO) enrichment using TRAPID.

**Figure 5:**
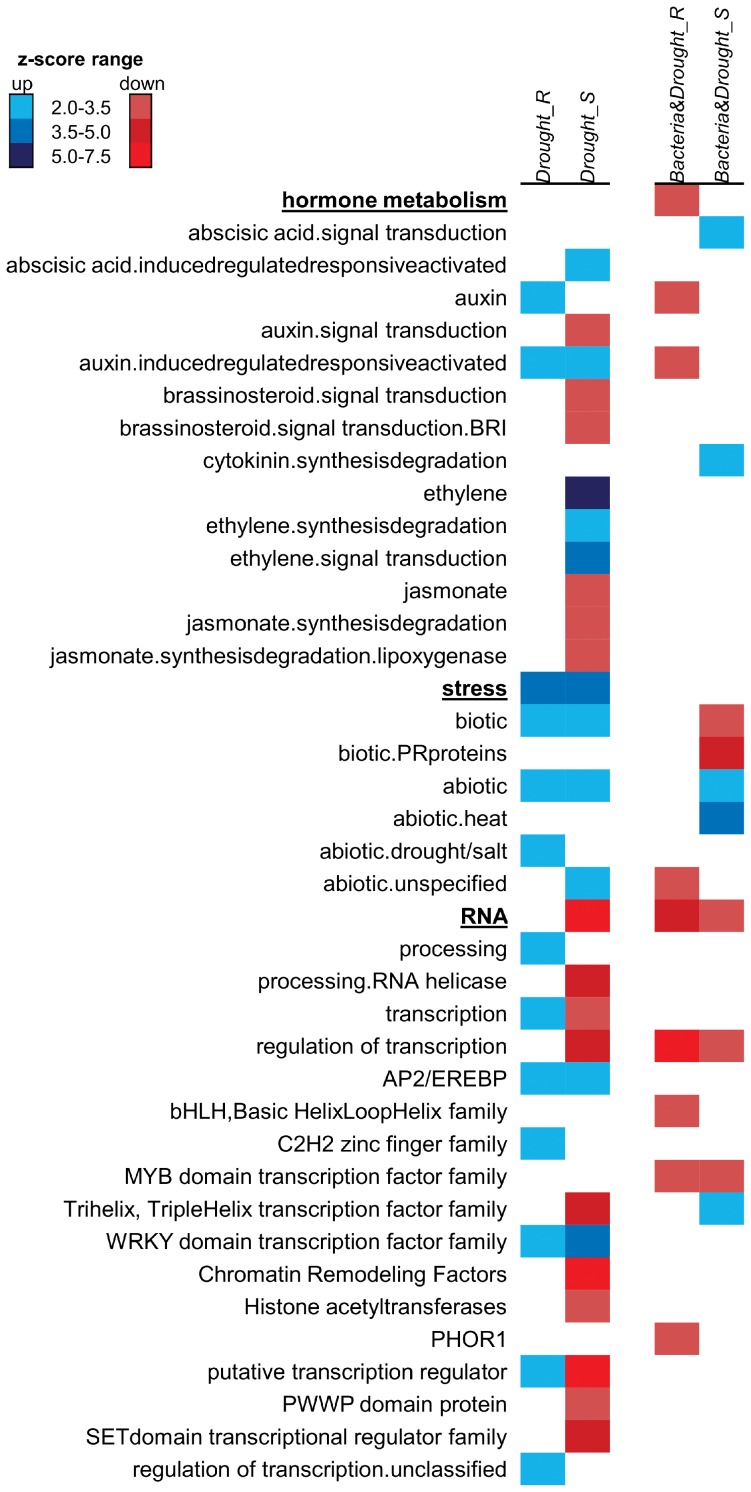
Gene set enrichment analysis of differentially expressed genes in sugarcane SP70-1143 colonized or not with *G. diazotrophicus* and subjected to water deficit. PageMan analysis of *Drought* and *Bacteria&Drought* dataset comparisons, for root and shoot tissues, using Bin-wise Wilcoxon test with Benjamini-Hochberg FDR multiple testing correction (ORA cutoff  = 1.0). Blue stands for upregulated/increased classes; Red stands for downregulated/decreased classes. For a complete overview, see Table S1.

Enrichment analysis of *Drought* DEG dataset using PageMan showed regulation of gene categories known to have an important role in drought responses (Figure 5), such as abiotic and salt/drought stresses; ABA and auxin hormones metabolism; as well as regulation of transcription involved in hormone signaling as AP2/EREBP and WRKY TFs that participate in ET and ABA-responsive signaling pathways, respectively [58], [59].

**Figure 6:**
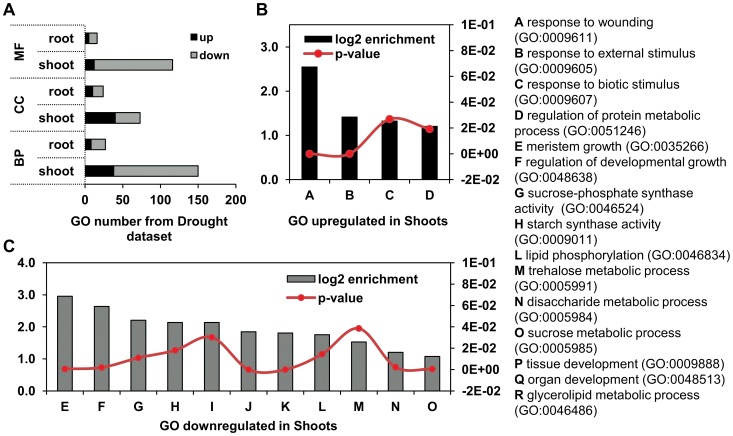
GO enrichment analysis of non-inoculated SP70-1143 plants under water deficit. TRAPID system calculated GO enrichment based on the *Drought* dataset compared to a background (p-value<0.05). (a) Number of GOs highly represented as up or downregulated, in shoots and roots. Graphs in (b) and (c) show enrichment level and p-value of selected GOs indicating drought pattern in shoots of *Drought* dataset, corroborating PageMan analysis (Table S1). GO enriched terms are shown as (b) up and (c) downregulated in shoots of SP70-1143. For a complete overview, see Table S2.

A search for enriched functional GO terms using the TRAPID web tool, in up- and downregulated DEG separately, found most GO terms differentially regulated in the shoot (Figure 6A), corroborating with the PageMan analysis. Functions as response to stimulus, transcriptional activity, calcium ion binding, regulation of cellular process and protein regulation were all increased in shoots of *Drought* dataset (Figure 6B). Remarkably, a great number of GO functions were repressed in both tissues, especially in shoots (Table S2). Those are most related to sugar metabolism and energy storage and important biological processes related to regulation of growth and development of meristems, tissues and organs (Figure 6C). This result supports evidences of plant growth restraining after 3 days under water deficit stress, especially in shoots [45], [46].

#### Hormone metabolism in the *Drought* dataset

**Figure 7:**
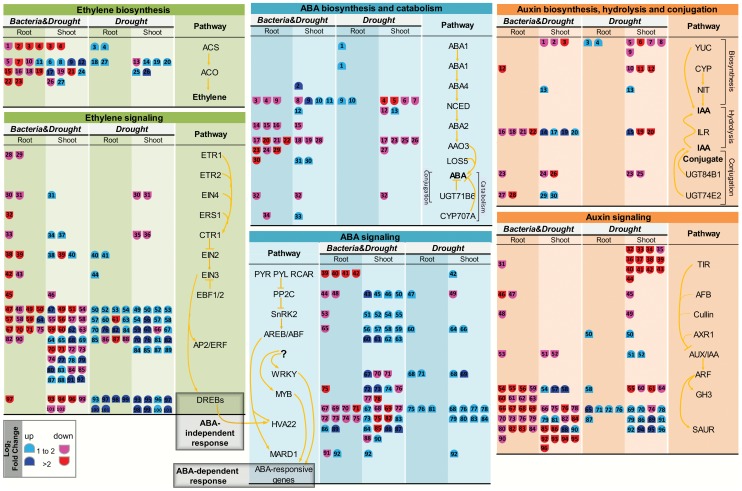
Hormonal responses to bacteria colonization and drought stress in sugarcane SP70-1143. Only differentially expressed genes (DEG) related to ABA, ET and auxin biosynthesis, signaling and response, present in *Drought* and *Bacteria&Drought* datasets, are indicated in the schemes of each hormone pathway. Expresssion levels (log2 fold change) are represented as colors and symbols. Red stands for downregulated and blue stands for upregulated genes. For each hormone pathway, the DEG are identified with numbers. For more detailed function of each regulated gene, see Table S3.

As recently reviewed by Bhargava and Sawant [24], drought response and adaption involve a complex and intricate biochemical machinery composed of different types of TFs and hormone-dependent regulation. The analysis of enriched gene functions and drought markers in the *Drought* dataset indicated that SP70-1143 might respond to drought both in ABA-dependent and -independent pathways. Gene enrichment analysis also identified ET and auxin responses as being increased in both roots and shoots from the *Drought* dataset. In order to characterize the hormonal response to drought stress of non-inoculated sugarcane SP70-1143, a more specific analysis of ABA, ET and auxin pathways was performed. Searching DEG on MapMan, differential expression patterns for biosynthesis, signaling and response to those hormones were identified. Different transcription patterns between shoot and root were observed. A simplified view of hormone metabolism can be seen in Figure 7. A Table listing log_2_ fold change of DEG of the three hormone pathways characterized in detail is presented in Table S3.

The data indicated an increase of ABA biosynthesis in roots under drought stress (Figure 7), since there was an upregulation of zeaxanthin epoxidase (ZEP/ABA1) and of a 9-cis-epoxycarotenoid dioxygenase family member (NCED3). ABA1 has a role in the first step of ABA biosynthesis; and NCED3 is a key gene in ABA biosynthesis that acts later on in the pathway catalyzing the carotenoid cleavage to form xanthoxin [60]. ABA signaling and response also seemed to be activated in roots (Figure 7), since members of ABA-insensitive (ABI) and AREB/ABF family of TF that regulate ABA responses were upregulated. In ABA-dependent responses, AREB/ABF proteins were the main *trans*-acting factors that bind the *cis*-element ABRE [23]. In contrast, ABA responses operating in SP70-1143 shoots under 3-days of water deficit were less clear. Several ABA signaling and response genes seemed to be activated in shoots, such as PYL8, a member of PYR1/PYL/RCAR family of ABA receptors, as well as two transcripts homologues to AREB3 and the drought molecular markers HAI3/PP2C, HVA22E and RD22A. ABA2 (encoding a zeaxanthin epoxidase) that is an intermediate of ABA biosynthesis was also upregulated in shoots. Nevertheless, several NCED family members were downregulated suggesting that ABA synthesis decreased in the shoots subjected to water deficit. This decrease was also supported by downregulation of several members of the ABA-aldehyde oxidase (AAO) gene family, including AAO3 which is known to catalyze the final step in abscisic acid biosynthesis in leaves, and downregulation of the ABA signaling gene ABI3 [61]. qRT-PCR analysis confirmed that PYL8, as well as HVA22F and RD22A were upregulated in shoots under water deficit (Figure 4A).Taking together, the data suggest that ABA signaling and response seem to be activated both in roots and shoots of non-inoculated SP70-1143 plants subjected to 3 days of water deficit, while ABA biosynthesis seems mostly activated in roots, compared to watered plants (Table S3).

ET biosynthesis appeared to be increased both in roots and shoots under drought stress (Figure 7). This was suggested by the upregulation of key genes in the pathway, such as several homologs of 1-aminocyclopropane-1-carboxylate oxidase (ACO) and, in roots, of 1-aminocyclopropane-1-carboxylic acid synthase 6 (ACS6), confirmed by qRT-PCR (Figure 4). ET synthesis begins with the formation of S-Adenosylmethionine (S-AdoMet) through methionine's catalysis by SAM synthetase. S-AdoMet can be converted to the immediate precursor of ET, ACC, by ACS. ACO acts on the final step on ET synthesis, using ACC as substrate. Both ACS and ACO are encoded by multigene families that can be regulated transcriptionally by biotic and abiotic stresses. Nonetheless, S-AdoMet can work as methyl group donor for nucleic acids, proteins and lipids and also as precursor in the polyamine synthesis pathway, which makes the conversion of S-AdoMet to ACC a crucial and rate-limiting step in ET biosynthesis [62]. In that case, high levels of ACS transcripts induced by stresses, such as drought, can be better associated with ET production [62], [63]. ET signal transduction and response also appeared to be increased in *Drought* datasets. This was shown especially by the downregulation, in shoots, of two transcripts with homology to the ethylene receptor EIN4, a negative regulator of ET signaling (Figure 7); and by the upregulation of several TFs involved in ET perception and signal transduction. Lower EIN4 mRNA levels were also identified by qRT-PCR (Figure 4A), corroborating the RNAseq data. In the signaling pathway most DEG belonged to the AP2/ERF superfamily of TFs, which suggests that ET responses were regulated in non-inoculated SP70-1143 under water deficit. At least 12 DEG classified as AP2/ERF TFs were equally upregulated in roots and shoots of SP70-1143 (Figure 7, Table S3). ERF proteins act primarily as regulators of transcription; having a possible role in responses to biotic and abiotic stresses as well as in development and metabolism [64]–[66]. In this work, members of the subfamily B1 of AP2/ERF, as ERF3, ERF7 and ERF12, known to be induced in response to water deprivation [67], [68], were upregulated in shoots and roots of the *Drought* dataset. A similar expression pattern was found for TINY2, a member of DREB family of TF, most present in leaves and known to be expressed in response to biotic and abiotic stresses [69]. The induced levels of expression of ERF12 and TINY under drought conditions were validated by qRT-PCR (Figure 4).downregulatedThus the results suggest that in root and shoot of non-inoculated SP70-1143 subjected to water deficit, ethylene biosynthesis, signaling and responses are activated.

Auxin biosynthesis and responses appeared to be activated in roots of non-inoculated SP70-1143 under water deficit. YUCCA genes, which encode an enzyme responsible for the synthesis of IAA from indole-3-pyruvic acid (IPyA) [70], were induced in root's *Drought* dataset. Members of the GH3 and SAUR families were also upregulated (Figure 7) and the increased mRNA levels of SAUR24 were confirmed by qRT-PCR (Figure 4B). It is already described that both GH3 and SAUR genes are auxin transcriptionally inducible genes [71], [72]. The GH3 family includes genes involved in auxin conjugation [71], and the function of SAUR-family genes is still unknown [72]. A greater number of genes involved in auxin biosynthesis, signaling and response were regulated in shoots of non-inoculated SP70-1143 under water deficit, compared with the auxin profile in roots. Auxin biosynthesis in shoots seemed to be mostly decreased during water deficit, which was suggested by the repression of YUCCA and CYP genes. Another route of auxin synthesis in plants is the indole-3-acetonitrile (IAOx) pathway. IAOx is produced through L-tryptophan by the action of several cytochrome P450s (CYP), and it is subsequently converted to indole-3-acetonitrile (IAN) [70]. This molecule is the substrate of the reaction catalyzed by nitrilases (NIT) leading to the production of the auxin IAA [70]. As shown in Figure 7, although one NIT2 gene was upregulated in SP70-1143 shoots, the CYP gene, responsible for the production of the substrate of the NIT reaction was downregulated. One important point of regulation of the auxin pathway is the auxin homeostasis, and this mechanism appeared to be operating in shoots under water deficit. Although plant hormone homeostasis depends on its biosynthesis, IAA conjugation with carbohydrates, amino acids or peptides contribute to this process, and IAA conjugate forms are generally considered inactive [70]. Conjugate hydrolysis is also a mechanism involved in the conversion to active auxin [70]. UDP glucosyltransferases (UGT) genes, which conjugate IAA to glucose, were downregulated in shoots, as well as most of the ILL/ILR genes, involved in hydrolysis of IAA-amino acid conjugates in *Drought* dataset [70]. The regulation of auxin signaling in SP70-1143 shoots after 3 days of water deficit was not so clear. While auxin receptors as AFB5 and ABP1 [71], negative regulators of the pathway, were downregulated, the auxin signaling repressor AUX/IAA31 [71] was induced, which could result in an inhibition of some shoot responses regulated by auxin. While GH3 family members were transcriptionally repressed in SP70-1143 shoots in response to water deficit, most of SAUR genes were induced. The increased mRNA levels of AUX/IAA31 and SAUR71 in shoots were confirmed by qRT-PCR (Figure 4A), The results indicate that shoots and roots present different profiles of regulation in SP70-1143 plants subjected to water deficit, and. auxin signaling is not very responsive to this stress in root. On the other hand, auxin biosynthesis, hydrolysis of auxin conjugates, as well as auxin signaling seem to be actively regulated in shoots under water deficit, being mainly repressed.

Altogether, analyses of DEG enrichment and drought molecular markers suggest that non-inoculated sugarcane cv. SP70-1143 responds to water deficit stress by the activation of well-known drought-responsive markers, as well as by the induction of hormone pathways well known to be involved in drought stress, such as ABA and ET. The decreased enrichment of GO terms related to development in shoots, together with downregulation of auxin pathway in shoots, suggest that one possible strategy adopted by sugarcane cv. SP70-1143 to try to tolerate drought might involve modulation of development, by restraining growth in shoots, while possibly improving growth in roots. This data is in accordance with the increased root to shoot ratio mostly seen in plants under drought conditions [45].

### Transcriptional profiling of sugarcane cv. SP70-1143 under water deficit and colonized with *G. diazotrophicus*

Analysis of differentially expressed pathways in the *Drought* dataset allowed to define the transcriptional response to drought stress of diazotrophic endophyte-free sugarcane SP70-1143. Another important question to be addressed is how *G. diazotrophicus* affects molecular and biochemical responses in SP70-1143 resulting in the observed improved tolerance to drought. To address this question, root and shoot transcriptome profiles of differentially expressed pathways in the *Bacteria&Drought* dataset were characterized and compared with *Drought* datasets using MapMan functional annotation and PageMan functional enrichment.

The most striking response of SP70-1143 plants under water deficit, when they were associated with *G. diazotrophicus*, was observed in roots. It was clearly represented in a MapMan visualization of regulatory processes, when comparing *Bacteria&Drought* and *Drought datasets* (Figure 3A,C). Remarkably, inoculated SP70-1143 roots submitted to 3 days of water deficit had a higher number of DEG downregulated when compared with non-inoculated roots in the same condition. On the other hand, this contrasting pattern of expression was not seen in shoots (Figure 3B, D).

Comparative analysis of differentially expressed pathways among the *Bacteria&Drought* and *Drought* datasets highlighted several gene classes with a very distinct pattern of regulation (Table S1 and Figure 5). There was an enrichment of DEG annotated to major classes responsive to abiotic stress such as stress responses and hormone metabolism, including TFs. Nevertheless, the PageMan functional enrichment demonstrated that the stress response genes, in general, had an opposite pattern of response between *Drought* and *Bacteria&Drought* datasets (Figure 5). They were strongly increased in both tissues of the *Drought* dataset, in contrast to the *Bacteria&Drought dataset*, which showed stress components only increased in shoots (Figure 5). Drought molecular markers previously identified as upregulated in *Drought* dataset were searched in *Bacteria&Drought*, in order to have a clear view on stress responses in this dataset. The dehydration responsive genes ERD1 and RD20A were upregulated in shoot both from *Drought* and *Bacteria&Drought* datasets (Figure 4A), which validated at the molecular level the experimental condition of water deficit. Notwithstanding, putative homologs to DREB1A/CBF3, DREB1B/CBF1 and NCED3 were downregulated when SP70-1143 was associated with *G. diazotrophicus* and submitted to 3 days of water deficit (Table S5). The opposite expression patterns of DREB1A/CBF3 in the two datasets were confirmed by qRT-PCR (Figure 4). The decrease in differentially regulated stress pathways suggest that some plant responses to drought might be inhibited in SP70-1143 colonized with *G. diazotrophicus*.

#### Hormone metabolism in the *Bacteria&Drought* dataset

In order to identify genes that could be directly or indirectly involved in the beneficial effects of *G. diazotrophicus* colonization conferring tolerance against drought, a more specific analysis of DEG belonging to the enriched hormone categories was performed. Expression patterns of crucial genes for synthesis, signaling and response to these hormones were comparatively analyzed in the two datasets.

PageMan comparative analysis between *Drought* and *Bacteria&Drought* datasets showed that most hormone classes were represented uniquely in shoots of the *Drought* dataset, as is the case for ethylene, jasmonate and brassinosteroids (Figure 5). Hormone enrichment for cytokinin metabolism was observed only in the *Bacteria&Drought* dataset. Auxin metabolism was enriched in both datasets, nevertheless with an opposite regulation. The ABA pathway was enriched in both datasets.

An opposite pattern of expression in both datasets was observed in roots for most of the DEG belonging to hormone pathways (Figure 7, Table S3). The biosynthesis and signaling of ABA, ET and auxin in roots of SP70-1143, colonized by *G. diazotrophicus* and subjected to water deficit, were mainly repressed. Contrasting with the expression profile in roots, in general, shoots from the *Bacteria&Drought* dataset did not show an opposite differential expression of hormone biosynthesis and signaling genes (Figure 7), suggesting that these hormone pathways might be induced or repressed depending on the tissue. Since the differences in the pattern of hormonal responses between *Bacteria&Drought and Drought* datasets were remarkable in roots, this organ was chosen to be investigated in more detail.

Analysis of DEG in the *Bacteria&Drought* dataset revealed a repression of ABA biosynthesis in roots of SP70-1143. This repression was indicated by the downregulation of positive regulators of ABA biosynthesis, such as NCED3, ABA2 and ABA3, and of several members of the AAO gene family, including the key element AAO3 [61]. ABA signaling also seemed repressed in roots in the *Bacteria&Drought* comparison, which was deduced from the downregulation of key regulators in almost every step in the ABA signal transduction, such as putative homologs of the ABA receptors (GCR2 and PYL8),), PP2C, SnRK2, AREBs and several HVA22 (Figure 7). qRT-PCR analysis confirmed that ABA2, PYL8 and HVA22F mRNA levels were downregulated in root or shoot of *Bacteria&*Drought dataset, and showed a different expression pattern compared to *Drought* datasets (Figure 4). The data suggests that in roots of SP70-1143 plants inoculated with *G. diazotrophicus* and under 3 days of water deficit, ABA biosynthesis, signaling and response are mostly inhibited when compared to non inoculated sugarcane in the same abiotic conditions (Table S3).

Members of ACS and ACO gene families, positive regulators of ET biosynthesis that are induced in the *Drought dataset*, were inhibited in *Bacteria&Drought* datasets (Figure 7). The reduced mRNA levels of ACS6 and ACO were confirmed by qRT-PCR (Figure 4). Negative regulators of ET signaling, such as ET receptors ETR1, ERS1 and EIN4 and Constitutive Triple Response 1 (CTR1), were repressed in roots, which could indicate an activation of the pathway. However, it has been shown that mRNA levels of ET receptors and CTR1 are increased in the presence of ET [73]. As ET synthesis was apparently reduced in *Bacteria&Drought*, it is possible that the lack of ET could cause a reduction of ET receptors in roots. Similarly, transcription of positive key regulators in ET signaling was also repressed in roots from *Bacteria&Drought*, suggesting that the ET pathway was not activated. This can be seen by the downregulation of the positive regulators EIN2 and EIN3-like, and ET response factors members as ERF1, ERF3 and ERF12, as well as members of DREB family of TF such as TINY2 and DREB1A/CBF3. The reduced levels of EIN4, EIN2, ERF12 and TINY were validated by qRT-PCR (Figure 4B). Remarkably, all the ET biosynthesis and response genes analyzed by qRT-PCR showed an opposite pattern of expression between *Drought and Bacteria&Drought* shoot datasets (Figure 4A). Thus, the inhibition of several ET responsive TFs, together with the reduction of biosynthesis, supports the hypothesis that the ET pathway is partially inhibited under drought stress when the sugarcane has been inoculated with *G. diazotrophicus* (Figure 7, Table S3).

In roots, all DEG annotated as members of the auxin pathway were repressed in the *Bacteria&Drought* dataset, including genes of auxin biosynthesis/homeostasis (CYP, UGT and ILL/ILR), signaling (AFB4, AFB2 and AUX/IAA31) and response (GH3 and SAUR) (Figure 7, Table S3). Interestingly, SAUR24 gene was the only DEG in root *Bacteria&Drought* dataset that was also regulated in the *Drought* dataset, although in an opposite manner, as validated by qRT-PCR analysis (Figure 4B). The opposite mRNA expression pattern of AUX/IAA31 and SAUR71 in the two treatments was also observed in shoots (Figure 4A) In general, the RNA-seq results suggest that while auxin signaling in roots is more activated in the *Drought* dataset, in the *Bacteria&Drought dataset* auxin biosynthesis, signaling and response are all downregulated (Figure 7). The opposite expression of auxin response genes SAUR24 and SAUR71 was also observed in qRT-PCR analysis (Figure 4B). Auxin has already been reported as a negative regulator of drought tolerance. In wheat, drought stress tolerance was accompanied by a decrease in IAA content [74]. Downregulation of IAA was found to facilitate the accumulation of late embryogenesis-abundant (LEA) mRNA, leading to drought stress adaptation in rice [75]. In this context, the results suggest that the inhibition of auxin pathway in roots could contribute to the higher level of drought tolerance observed in *G. diazotrophicus* inoculated SP70-1143 plants.

The results showed that while SP70-1143 non-inoculated plants under water deficit activate well known responses to drought stress, the association with *G. diazotrophicus* leads to an opposite expression profile in roots by suppressing several of the drought stress genes. This data suggests that colonized roots would not be suffering from stress in the same way as non-inoculated plants. An important issue to be addressed is which gene and pathways are directly involved in mechanisms that regulate the tolerance to drought conferred by *G. diazotrophicus*.

### Transcription Factors in ABA-dependent and ABA-independent response to drought

The various hormonal plant responses to stresses trigger changes in gene expression that are coordinated by the action of TFs, culminating in the regulation of different physiological and biochemical responses [22]–[24]. Some major gene families of drought responsive TF were highly regulated in both datasets. Among them were members of ABA-dependent and independent response to drought, such as the AP2/ERF superfamily that is known to be responsive to drought mostly in an ABA-independent manner [51], and the AREB/bZIP, WRKY and MYB gene families that are important TFs in the ABA-dependent drought response [76]–[78].

**Figure 8:**
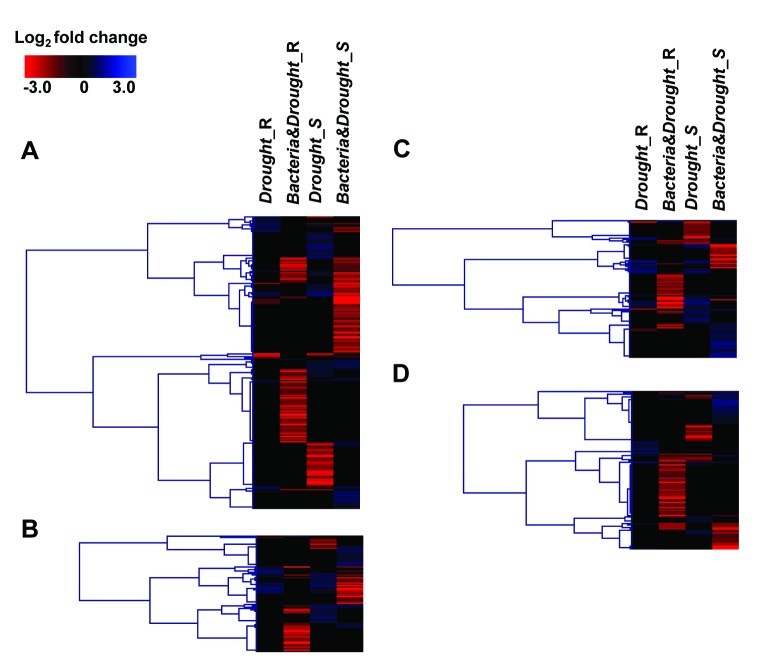
Hierarchical clustering of drought responsive transcription factors in cv. SP70-1143. Expression pattern of (a) MYB, (b) WRKY, (c) ERF/AP2 and (d) bZIP gene families, in both root and shoot of sugarcane *Drought* and *Bacteria&Drought* datasets. Both genes and datasets were clustered in Genesis software, using Pearson correlation and Average linkage. For specific analysis of each gene after hierarchical clustering, see Table S4.

In order to analyze how these TFs behave in the investigated experimental conditions, DEG belonging to these gene families were grouped using hierarchical clustering (Figure 8, Table S4). In each gene family, there was more upregulated DEG than downregulated, in both roots and shoots of non-inoculated SP70-1143 under water deficit (Figure 8). Together with validation of the differential expression of RD20A, ERD1, HVA22F and DREB1A/CBF3, the data suggest an activation of both ABA-dependent and independent responses in diazotrophic-free SP70-1143 under water deficit. In contrast, the expression profile of MYB, WRKY, bZIP and AP2/ERF genes were mainly inversely regulated in the *Bacteria&Drought* dataset compared to the *Drought* dataset (Figure 8). In roots, it was remarkable that most TFs were downregulated in SP70-1143 plants under drought stress when inoculated with *G. diazotrophicus*. Nevertheless, in shoots some classes of these TF families were found upregulated in *Bacteria&Drought* dataset, and they represented mainly genes different from the ones upregulated in the *Drought* dataset. Therefore, these TF datasets were searched for expression profiles that could help to identify mechanisms specifically activated by *G. diazotrophicus* that would be involved in regulating tolerance to drought stress in SP70-1143 plants.

Among the 221 differentially expressed transcripts annotated as members of MYB family of TFs, 28 were upregulated in shoots of inoculated plants. In contrast, the same TFs were generally not DE in roots, with the exception of MYB19 and MYB70, that are downregulated (Figure 8, Table S4). Some studies with members of MYB-family have been shown that they play essential roles in abiotic stress responses in ABA-dependent manner [79]–[81]. It was noticeable that 15 transcripts annotated as MYB-type TFs were differentially expressed exclusively in the *Bacteria&Drought data*sets, wherein 13 were up-regulated only in shoots and the remaining 2 transcripts were downregulated in roots. It corroborates with the up-regulation of ABA biosynthesis genes observed exclusively in shoots of inoculated SP70-1143 plants under drought stress (Figure 7). Remarkably, MYB108 was the most induced transcript in inoculated SP70-1143 submitted to drought. In *Arabidopsis*, MYB108 was described acting together with MYB24 to regulate Jasmonate-mediated stamen maturation and pollen viability [82].This data can suggest a new role for this gene, acting in the mechanism of drought resistance conferred by an endophytic bacteria. Another up-regulated transcript in the *Bacteria&Drought shoot data*set, was annotated as MYB44. The overexpression of this gene in *Arabidopsis* enhances ABA-induced stomatal closure conferring abiotic stress tolerance [80]. Contrastingly, a transcript annotated as *AtMYB61*, that is an ABA-independent regulator of stomatal closure in *Arabidopsis* [83], was downregulated.

WRKY TFs are involved in many biological processes in plants, including seed dormancy and germination, senescence, biotic stress and, most recently, many studies have unraveled their roles in abiotic stress responses [59], [84]. Interestingly, it has been reported the presence of ABRE cis-elements in the promoter region of *WRKY* genes [85]. *WRKY2* T-DNA insertion mutants were hypersensitive to ABA in germination and postgermination of *Arabidopsis* [86]. Notably, *WRKY2* was the highest upregulated transcript exclusively expressed in shoots of inoculated SP70-1143 plants submitted to drought. These data may provide an insight of a negative feedback in the ABA-signaling pathway through WRKY TFs, indicating that a fine-tuning regulation is necessary in inoculated SP70-1143 plants coping with drought. Contrastingly, *WRKY18* and *WRKY40* that are negative regulators of ABA-signaling [87], were induced only in non-inoculated plants, suggesting that part of the ABA responses mediated by WRKY TFs might be activated when plants under drought stress are colonized with *G. diazotrophicus* (Figure 8,Table S4).

In the DE datasets, 120 transcripts were annotated as members of bZIP family of TFs (Figure 8, Table S4). Among the bZIP TFs, AREBs are assigned as group A and have roles during drought and high-salinity conditions in an ABA-dependent pathway [78], [88]. Most transcripts annotated as AREB1 and AREB2 were only upregulated during drought in shoots of inoculated plants, whereas AREB3 was only upregulated during drought in shoots of non-inoculated plants. ABF3 that also belongs to group A of bZIP TFs was upregulated in shoots of inoculated SP70-1143. Interestingly, two transcripts annotated as FD-1, another group-A bZIP, whose mutants in Arabidopsis are late flowering, were downregulated in roots of inoculated plants [89], suggesting that these plants are not suffering from drought when *G. diazotrophicus* is in the scene. Together, these results suggest that AREB1, AREB2 and ABF3 could act as key elements in the drought resistance conferred by *G. diazotrophicus* to SP70-1143.

Among the Ethylene Responsive Factor family of TFs (ERF), a set of genes from subfamily known as DREB (Drought-responsive element binding) is induced to help plants to cope with abiotic stresses including drought and cold. It occurs in an ABA-independent manner through binding to the DRE/CRT cis-acting elements upstream of stress-related genes [90]. Formerly, DREB1A was recognized to be involved in the signal transduction pathway responsive to low temperature [91], but more recently many studies have demonstrated DREB1A playing a role in increasing salinity and drought tolerance in plants [92]–[95]. DREB2A is thought be a major transcription factor that functions under dehydration in high-salinity conditions [90], [96]. Recently, it was shown that DREB2A could confer drought resistance to a sugarcane transgenic plant [97]. Some transcripts annotated as DREB1A and DREB1B were upregulated in SP70-1143 plants submitted to drought (Figure 4 and 7) while DREB2A was not responsive to this condition. Unexpectedly DREB1A, DREB1B and DREB2A were downregulated when SP70-1143 plants submitted to water depletion were inoculated *with G. diazotrophicus*, suggesting that the resistance to drought conferred by the diazotrophic bacteria does not depend on DREB1/2 downstream mechanisms. Interestingly, two transcripts annotated as *RAP2.6L* were highly induced exclusively in shoots of inoculated SP70-1143 (Figure 8, Table S4). RAP2.6L belongs to the ERF subfamily of AP2/ERF and studies in *Arabidopsis* demonstrated its responsiveness to ABA treatment [98]. All together, the data suggests that the ABA-independent pathway is not active when SP70-1143 is colonized by *G.diazotrophicus* under water depletion.

The results on the comparison of hormonal responses between *Bacteria&Drought* and *Drought* datasets showed that SP70-1143 responds in a classical way to water deficit, activating well-known drought-responsive markers, as well as inducing ABA-dependent and independent pathways that are well known to promote responses to drought stress. Nevertheless, the association with *G. diazotrophicus*, which increases drought tolerance in SP0-1143, suppresses several of these drought stress responses in roots. On the other hand, shoots from inoculated plants under drought conditions specifically activate some TFs that participate in ABA-dependent signaling, and could act as key elements in the drought resistance conferred by *G. diazotrophicus* to SP70-1143. Besides, the downregulation of auxin pathway during the early responses to water deficit in cv. SP70-1143 colonized by *G. diazotrophicus* could participate to help plants to tolerate prolonged water deficit.

## Conclusions

Sugarcane is naturally colonized by diverse types of diazotrophic bacteria and non-nitrogen-fixating organisms that can interact between each other and the host plant. In the present work, the study of the interaction of sugarcane cv SP70-1143 with the diazotrophic bacteria, *G. diazotrophicus*, provided first insights of how beneficial associations with nitrogen-fixating bacteria could affect plant metabolism to improve stress response and survival of host plants.The results showed, for the first time, that *G. diazotrophicus* can improve drought tolerance in sugarcane cv. SP70-1143, prolonging its survival even after 40 days withholding water. RNA-seq data, corroborated by qRT-PCR expression analysis, showed that several molecular and biochemical responses to water deficit were differentially regulated when plants were colonized by the beneficial diazotrophic bacteria compared to plants free of bacteria. This was clearly shown by the opposite ABA and ET signal transduction responses to drought, mainly downregulated in SP70-1143 inoculated plants and upregulated without the bacteria's presence. We can hypothesize that SP70-1143 has its early drought responses partially inhibited because *G. diazotrophicus* might be softening plant stress in several aspects. Thus, the divergent transcriptome profile in *Bacteria&Drought* compared to *Drought* dataset could reflect gene expression in a less stressed plant.

A major challenge is to unravel the mechanisms regulated by colonization with *G. diazotrophicus* that are in fact mediating the drought tolerance conferred to SP70-1143. Transcriptomic and physiological data suggest that SP70-1143 might modulate development, restraining shoot growth while possibly improving root growth, in order to overcome stress; but not when associated with *G. diazotrophicus*. Downregulation of the complete auxin pathway in roots when the diazotrophic bacteria are present supports this hypothesis, since this hormone is a positive regulator of root growth. Also, colonization with *G. diazotrophicus* seems to shift SP70-1143 gene expression during drought stress to specific ABA-dependent responses. TFs specifically activated in shoots from inoculated plants under drought conditions, which participate in ABA-dependent signaling, were identified and could act as key elements in the drought resistance conferred by *G. diazotrophicus* to SP70-1143.

The present work describes the initial events that may trigger the sugarcane drought tolerance promoted by *G.diazotrophicus* inoculation. In the future, a clear understanding of the mechanisms of drought tolerance during plant interaction with diazotrophic bacteria could provide tools to maximize the benefits for crop production.

## Materials and Methods

### Water deficit assay

In order to acquire microorganism-free plants, sugarcane plantlets (*Saccharum spp.* cv. SP70-1143) obtained by sterile meristem culture and kindly provided by CTC (Centro de Tecnologia Canavieira) (Piracicaba, São Paulo, Brazil) were cultivated *in vitro* in Murashige and Skoog medium [99] supplemented with sucrose (2%), citric acid (150 mg/L), kinetin (0,1 mg/L) and 6-BA (0,2 mg/L) or IBA (0,2 mg/L) for multiplication and rooting, respectively. *In vitro*-grown sugarcane plantlets were maintained in a 12 h light-dark cycle, at 28°C. After the development of a root system, the almost one month old plantlets were transferred to a hydroponic system in plastic containers (16 liters) supplemented with 1× Hoagland's solution [100]. Plantlets were acclimated during 14 days in a greenhouse at 28°C and then they were submitted to a Hoagland solution without nitrogen during 7 days before co-cultivation with the diazotrophic bacterium *Gluconacetobacter diazotrophicus* strain PAL5 for another 7 days. Non-inoculated control plants were grown in the same conditions. After this inoculation period, plants were transferred to pots (5 L) with mixed sand and vermiculite, at 2∶1 ratio and acclimatized for one week, where they were normally watered at two-day intervals, and then divided in four groups: i) non-inoculated, normally watered (CT) at two-day intervals; ii) non-inoculated, submitted to water depletion (WD); iii) inoculated, normally watered (GD) at two-day intervals; iv) inoculated, submitted to water depletion (WD+GD). In these conditions, plants were grown in a greenhouse at 28°C. Roots and shoots were harvested separately at 3 and 7 days after water deficit. For every condition, plant material from two pots was harvested. Each pot with three plants each represented one biological replicate and all tree plants, separately into roots and shoots, were pooled for RNA extraction. One of the replicates, at day 3, was used in the construction of RNA-seq libraries, generating a total of eight libraries. To observe the impact on plant phenotype, a third biological replicate of each condition was grown for 40 days forward.

To prepare the bacterial inoculation solution, the *G. diazotrophicus* strain PAL5 was grown on a Petri dish with LGI-P solid medium (sucrose or sugar 100 g/L, K_2_HPO_4_ 0.2 g/L, KH_2_PO_4_ 0.6 g/L, MgSO_4_.2H_2_O 0.2 g/L, CaCl_2_.2H_2_O 0.02 g/L, Na_2_Mo_4_.2H_2_O 0.002 g/L, Bromothymol blue 5 mL/L (0.5% solution in KOH 0.2N), FeCl_3_.6H_2_O 0.01 g/L, pH 5.5–6.0). Bacterial suspensions from one colony were grown in 5 mL liquid DYGS (glucose 2 g/L, peptone 1.5 g/L, yeast extract 2 g/L, K_2_HPO_4_ 0.5 g/L, MgSO_4_.7H_2_O 0.5 g/L and glutamic acid 1.5 g/L, pH 6.0) for two days at 28–30°C with agitation (120 rpm) as a pre-inoculum. To make the inoculation solution, 1 mL from pre-inoculum was added to each 100 mL liquid DYGS and grown overnight at 28–30°C (120 rpm). Bacterial suspensions with OD_600nm_ equal to one were added to 1× Hoagland's solution at a proportion of 1∶30. The same amount of medium without bacteria was added in Hoagland's solution as mock controls. *G. diazotrophicus* PAL5 was kindly provided by EMBRAPA, Seropédica, RJ.

The fresh weights (FW) of roots and shoots from 8 to 10 plants were measured 1 day before the beginning of stress. Statistical analyses were performed using One-way analysis of variance, followed by Bonferroni's Multiple Comparison Test.

### RNA isolation, RNA-sequencing and quality control

Material from three plants was collected per condition, immediately frozen in liquid nitrogen, and ground to minimize the effect of transcriptome variability among individual plants. Total RNA was isolated from root and shoot samples using Trizol (Invitrogen, CA, USA) as described by the manufacturer. The amount of RNA was measured using a Thermo Scientific NanoDrop 2000c Spectrophotometer and the quality was verified by electrophoresis on a 1% agarose gel with ethidium bromide staining. Total RNA (20 µg) from eight samples (shoot and root from CT, WD, GD and WD+GD) was sent to Fasteris Life Sciences SA (Plan-les-Ouates, Switzerland) for construction of mRNA libraries and deep sequencing on a HiSeq 2000 system (Illumina) using a single-end 100 cycle protocol. Demultiplexing was used prior to generation of fastq sequence files by separating the libraries according to their indexes. Raw reads in the fastq format were cleaned using quality trimming and quality filtering as implemented in the FASTX Toolkit (version 0.0.13, http://hannonlab.cshl.edu/fastx_toolkit/). For quality trimming, a quality threshold of 20 was used with a minimum read length of 20 nucleotides. For quality filtering, the minimum quality score was set to 20 in a minimum percent of bases of 90%. The sequence data from this study have been submitted to the NCBI Sequence Read Archive (http://www.ncbi.nlm.nih.gov/Traces/sra/sra.cgi) under accession no. SRP043291.

### *De novo* transcriptome assembly, read mapping and counting

Two assemblies were performed, the first one using the sequencing result from a previous sugarcane reference transcriptome build in-house, the second one using the sequencing result of the samples for differential expression analysis. For both assemblies, VELVET (version 1.0.12) [37] was used in combination with OASES (version 0.1.15) [38]. Several values of “kmer” (35–49 and 24–41 in the first and second assembly respectively, only odd numbers allowed) were used to optimize the assembly process. The average insert size was set to 200 bases with a standard deviation of 10%. A minimum size of 100 bases was set for the contigs and the coverage cutoff for contigs was set to 6X. In order to construct a final reference transcriptome, both assemblies were combined. To avoid redundancy, CD-HIT (version 4.6) [39] was used to detect sequences present in both assemblies with a sequence similarity threshold of 0.95. The final reference transcriptome consisted of all the sequences from the first assembly and representative sequences from CD-HIT clusters (sequence similarity threshold of 0.95) of sequences from the second assembly that were not found in the first assembly. To align the reads to the reference transcriptome, the Burrows-Wheeler Aligner (BWA, version 0.7.5a-r405) [42] was used with default parameters. Reads that aligned with too many mismatches were discarded. Here, reads with length 17, 38, 64, 93, 124, 157, 190 and 225 were discarded if they aligned with more than 2, 3, 4, 5, 6, 7, 8 and 9 mismatches respectively. Read mapping was performed at the transcript level and read counting was performed at the locus level. The read count for a given locus was obtained by summing up all the reads that aligned to each of the transcripts of that locus.

To measure the representation in the RT2 of plant or *G. diazotrophicus* loci, a BLASTX, with an e-value cutoff of 1e-5, was done against the PLAZA 2.5 proteome database (Viridiplantae) and a BLASTN was done against 7564 *G. diazotrophicus* coding sequences downloaded from: http://www.ebi.ac.uk/ena/data/view/Taxon:33996 (e-value cutoff 1e-5, %ID≧95 and minimum length of the alignment ≧50% of the sugarcane sequence).

### Differential expression analysis and functional characterization

To select differential expressed loci, read counts were normalized as RPKM (Reads Per Kilobase of transcript per Million mapped reads). For each sugarcane putative gene, in each library, RPKM was obtained dividing the transcript read count by the transcript length and by the total read counts, and multiplied by 1 billion [101]. This gave counts comparable between the samples. A Fisher's Exact test with a p-value cutoff <0.05 was performed on every combination of the 8 libraries using online version of IDEG6 [102] and default parameters. A Log_2_ Fold change (Log_2_FC) was used to create a transcriptome dataset with comparisons of interest. The fold change was calculated dividing the RPKMs from a condition of interest by the control, to measure the difference of expression between control and the condition of interest. For some of the genes, read counts were present only in one of the two libraries in each pairwise comparison, making impossible to quantify the Log_2_FC. Those loci with p<0.05 were called “exclusive” and a representative value of 1 or −1 was used as Log_2_FC. As a second cutoff, only those genes with |Log_2_FC|≧1, a difference of expression at least 2-fold higher or lower than the control, were selected as differentially expressed. To better select the genes related to plant response to water deficit only when associated with bacteria, loci that had an overlap between *Bacteria&Drought* and *Drought* or *Bacteria* comparisons, for shoot or root tissues, were removed from *Bacteria&Drought* datasets using a Venn Diagram (http://bioinformatics.psb.ugent.be/webtools/Venn/), leaving a dataset of genes uniquely regulated by bacteria during water deficit.

To assign a function to each sugarcane gene, RT2 sequences were searched against *Arabidopsis thaliana* and *Oryza sativa* proteins databases and best BLAST hits were retained (E-value<1e-05). A MapMan reference mapping was created with *A. thaliana* bincodes and using the *O. sativa* genomic database to complement the functional annotation. This mapping was uploaded on the MapMan platform, together with lists of DEG, in order to visualize diagrams of metabolic pathways or other processes.

Functions of differentially expressed genes (as Arabidopsis or Rice homologous) from both pairwise comparisons were visualized using MapMan [44]. Gene set enrichment analysis was performed using the PageMan visualization tool [103] with Bin-wise Wilcoxon test, Benjamini-Hochberg FDR multiple testing correction and 1.0 as ORA cutoff. TRAPID [43] was used to assign annotations and Gene Ontology (GO) terms to the predicted genes of Sugarcane SP70-1143. TRAPID was also used to detect open reading frames and frameshift correction in each transcript, which was useful to design specific sugarcane primers. The final Sugarcane Reference Transcriptome (RT2) was loaded to the TRAPID database, which uses the PLAZA 2.5 database [40], available at http://bioinformatics.psb.ugent.be/plaza/, to assign functions based on sequence similarity. If the length of a transcript was not strongly different than the average protein length of the gene family it was assigned to, it received the label “Quasi Full Length” as meta-annotation. When a transcript was assigned as “Quasi Full Length”, and its associated ORF had both a start and stop codon, than the meta annotation was changed to “Full Length”. To add gene families and functional annotations to each transcript, the sequences from the final RT were processed using the following pipeline for similarity searches: “phylogenetic clades”, “monocots” (database type), 10e-2 (e-value), “gene families” (gene family type) and “transfer from both gene family and best hit” (functional annotation type). DEG from each comparison (*Drought* and *Bacteria&Drought* for shoot and root) were set as subsets on TRAPID to distinguish them in the entire RT. GO enrichment analysis was done based on the dataset compared to a background (p-value<0.05). Hierarchical clustering was done using Genesis software (version 1.6.0 beta1), with Pearson correlation and Average linkage clustering (between genes and experiments) [104].

### RNA expression by qRT-PCR

Total RNA isolated from roots and shoots were treated with DNAse I (Biolabs). Reverse transcription was made using Super-Script III reverse transcriptase (Invitrogen) and random hexamers as primer, according to the manufacturers instruction. To analyze gene expression, qRT-PCR reactions were performed with SYBR Green PCR Master Mix (Applied Biosystems). To each well, 2.5 µL of 1× diluted first strand cDNA, 5 µL of SYBR Green solution, 0.9 µL of the forward primer (10 µM) and 0.9 µL of reverse primer (10 µM) were added, along with 0,7 µL of sterile, ultrapure water to bring the final volume to 10 µL in each well. qRT-PCR was performed using Applied Biosystems 7500 Real-Time PCR Systems, under standard conditions. The constitutive plant 28S ribosomal RNA (28S rRNA) was used as reference gene. To confirm bacteria colonization in plant inoculated tissues, specific primers were designed for amplification of *G. diazotrophicus* 23S rRNA. To validate the expression pattern of differentially expressed transcripts identified in the RNA-seq analysis, 15 specific primers were designed with Primer Express software (Table S6). The sequence used for primer design of each transcript was carefully selected in order to be specific to each putative gene. To accomplish that, the sequence of interest was aligned to the NCBI sugarcane database, to the currently available sugarcane reference transcriptome, and to other sugarcane transcripts annotated with the same function in *A. thaliana* or *O. sativa*. Only sugarcane transcripts with specific regions were selected for validation. For each sample (a pool of three plants), reactions were performed with three technical replicates, and with two biological replicates. The results of qRT-PCR were analyzed using ΔCt quantitative method according to Livak and Schimittgen [105]. Statistical analyses were performed using unpaired t-test.

## Supporting Information

Figure S1 **Identification of DEG in** ***Bacteria&Drought*** **root and shoot datasets by Venn diagrams.** The loci equally regulated by the pairwise comparisons GD *vs* CT and WD+GD *vs* WD (A, B), and WD *vs* CT and WD+GD *vs* WD (C, D) were subtracted from WD+GD *vs* WD comparison to generate the *Bacteria&Drought* datasets, which are indicated by arrows.
(TIF)

Figure S2 **New assembly contribution to transcriptome differential expression analysis.** Percentage of genes, from both assemblies R1 and R2, that were annotated in each MapMan category. The percentages were calculated based on the total reference transcriptome RT2 (R1+R2). Blue arrows indicated the major R2 contribution.
(PDF)

Table S1 **Pageman gene set enrichment analysis using Bin-wise Wilcoxon test with Benjamini-Hochberg FDR multiple testing correction (ora cutoff  = 1.0).**
(XLSX)

Table S2 **Complete GO enrichment analysis processed by TRAPID system in root and shoot of** ***Drought*** **dataset.**
(XLSX)

Table S3 **Differentially expressed genes in SP70-1143 (a) ethylene, (b) abscisic acid and (c) auxin metabolism as analyzed by MapMan.** Log_2_ fold changes for two pairwise comparisons for each of the experimental conditions, *Bacteria&Drought* and *Drought*, are indicated for shoot and root tissues.
(XLSX)

Table S4 **Hierarchical clustering results of (a) MYB, (b) WRKY, (c) ERF/AP2 and (d) bZIP gene families among root and shoot of** ***Drought*** **and** ***Bacteria&Drought*** **datasets.** Log_2_ fold changes are shown. Genes marked in yellow were the ones indicated in the text as involved with drought responses.
(XLSX)

Table S5 **Drought molecular markers annotated in** ***Drought*** **and** ***Bacteria&Drought*** **datasets.**
(XLSX)

Table S6 **Selected genes for qRT-PCR validation, primer sequences and Log_2_ fold changes in** ***Drought*** **and** ***Bacteria&Drought*** **datasets, for both root and shoot tissues.**
(XLSX)
