# Supplementary material for: Drought Tolerance Conferred to Sugarcane by Association with Gluconacetobacter diazotrophicus: A Transcriptomic View of Hormone Pathways
Source: PLoS One. 2014 Dec 9;9(12):e114744. doi: 10.1371/journal.pone.0114744 (PMC4260876; doi:10.1371/journal.pone.0114744)
Supplement: Figure S2 — New assembly contribution to transcriptome differential expression analysis. Percentage of genes, from both assemblies R1 and R2, that were annotated in each MapMan category. The percentages were calculated based on the total reference transcriptome RT2 (R1+R2). Blue arrows indicated the major R2 contribution. (PDF) [file pone.0114744.s002.pdf]

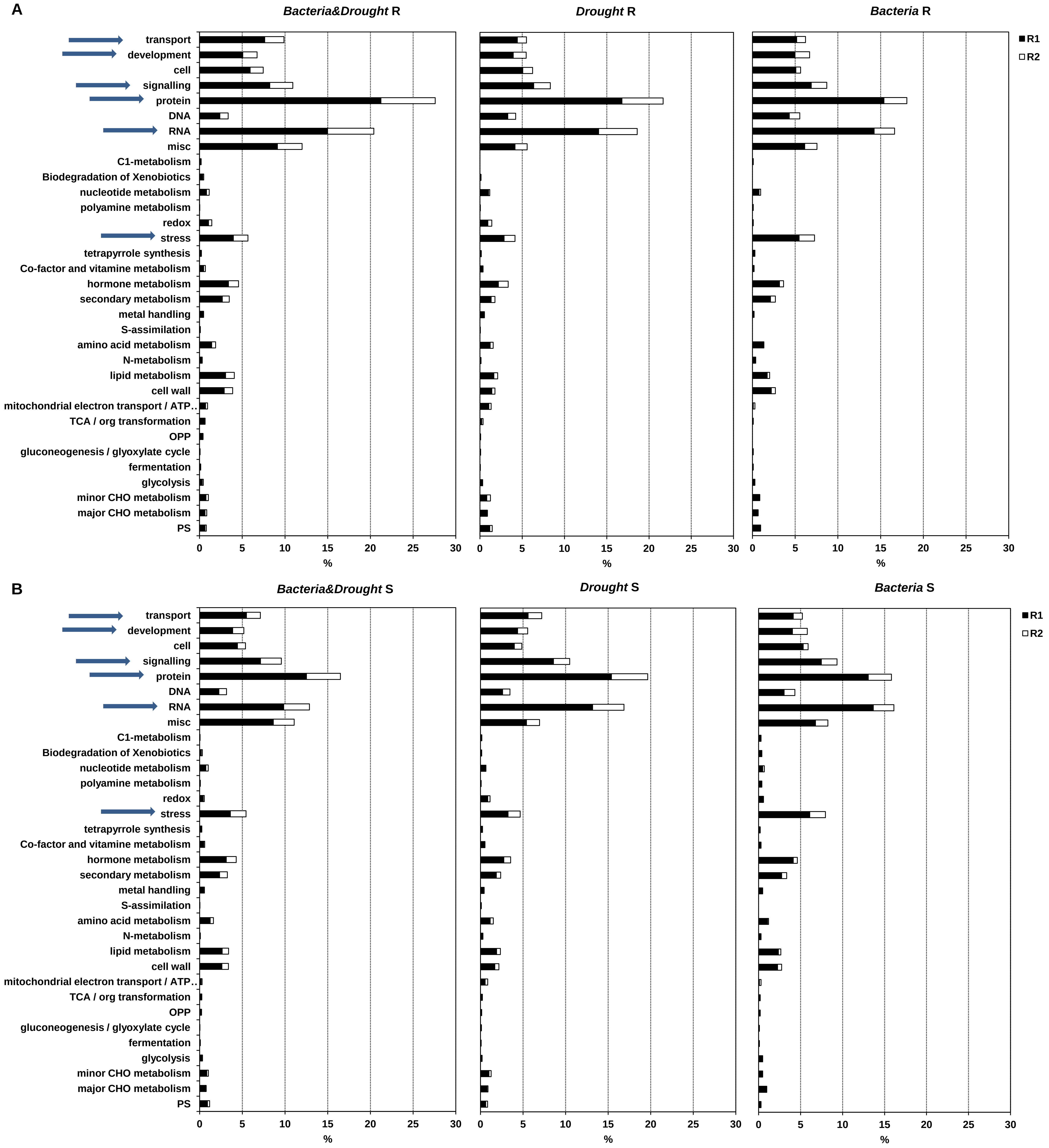

Figure S1. New assembly contribution to transcriptome differential expression analysis.
Percentage of genes, from both assemblies R1 and R2, that were annotated in each MapMan category. The percentages were calculated based on the total reference transcriptome RT2 (R1+R2). Blue arrows indicated the major R2 contribution.
